# Surgical Society of Ireland

**Published:** 1846-04

**Authors:** 


*Surgical Society of Ireland.*—At a meeting of the Society held in Dublin, Nov. 29th, 1845, Dr. Beatty said he was about to bring under the notice of the society a case which was possessed of considerable interest in many points of view. The specimen before them was a monster, which had been sent up for exhibition by Dr. Purefoy, of Cloughjordan, the account given being as follows:—It was one of four children, born of the same mother at a single birth, three of whom were born alive and healthy. The first child was born at ten o'clock at night; in fourteen hours after a second appeared; in an hour after this monster came forth; and in an hour after that, the fourth, a living child, was born. The only cause assigned for the occurrence was, that the mother had suffered considerable mental distress at about the seventh month of her pregnancy, from having seen the mutilated corpse of a cousin who was murdered in the neighbourhood; to this circumstance the neighbours were disposed to attribute the occurrence in question. The case was remarkable (Dr. Beatty observed) first, from the plurality of children; secondly, the birth of three alive, and in perfect condition; and thirdly, from the supposed cause of the occurrence which he had just mentioned. No examination had as yet been made of the preparation, as he was anxious to present it in as perfect a form as possible. It appeared to be, he said, one of those acephalous monsters, with, in addition, considerable deformity of all the limbs. Looking in the situation of the vertex, traces of the remains of a skull could be observed, and the specimen was found to differ from the ordinary acephalous monsters in their being no face. A slight projection occupied the natural situation of the nose, but no trace of any other feature existed. The upper limbs were particularly distorted, the arm being exceedingly short, and an absence apparently of the forearm, there being at the extremity of the arm a fin-like process representing a hand; the body, thorax, and pelvis appeared pretty natural, though very much misshapen; club feet were attached to both lower extremities; there was no trace of an anus; the dorsal region presented nothing unusual, there being no appearance of *spina bifida.* The monster was of the male sex. While thus examining the specimen, Dr. Beatty observed, the subject of monstrosity at large seemed to suggest itself for consideration, so that a few observations on that question might not be inapplicable just then. All present were no doubt aware, he said, that at the present moment the subject of monstrosities has given rise to a great deal of speculation in the minds of inquiring persons, leading to differences of opinion as to classes and kinds, some founding a classification arising from a redundancy of parts, as in children born with supernumerary fingers and toes, an occurrence by no means common; others, again, founded a classification depending on deficiencies of parts, for instance, of the arms, or legs, or the absence of the hand, and soforth. Again, monsters were to be found with all the parts apparently present, but in a misshapen condition, the arms and legs being both perhaps pretty naturally formed, but short and deficient in size, &c. The cases presenting a deficiency of all the parts had been classified as conglomerate or mixed prodigies, their being apparent only a mass of flesh, and to this class of cases he would call their attention presently. A very common cause of monstrosity, he would remind them, was a deficiency of the upper part of the calvarium, called cat-headed, from the resemblance to that animal. This, he observed, would appear to be the case in the fœtus under examination, but presenting, in addition, an example of deficiency and of misshapen parts. A question with many has been the cause of the deficiency of the calvarium in these cases, and among a great many opinions offered, it appeared to him that the one now put forward by Rudolphi of Berlin, was nearest the truth. Rudolphi is of opinion that in the ordinary acephalous fœtus the deficiency of the brain is attributable to the fœtus in utero having got hydrocephalus, the fluid in which continues to increase till it bursts through the brain and its investing membranes, and finally through the integuments, the fœtus escaping with life, but born without the brain, and with complete absence of the upper part of the head. In the museum at Berlin, are numerous specimens prepared by Rudolphi, exhibiting the disease in all its stages,—one with the head so enlarged by fluid as to be on the point of bursting; another, with a projection through the integuments of a vesicular character, the covering being a very delicate membrane through which the contained fluid is seen, and the brain, apparently, totally destroyed. From these and other proofs, Dr. Beatty considered that we are justified in accepting Rudolphi's explanation of the occurrence of acephalous fœtuses as the one most likely to be correct. Now, with respect to mental impressions, he would observe, that this is a vexed question, and has been for many years; however, numerous instances on record go to show that some connection exists between impressions made upon the mother and the fœtus in utero. He would not of course detain them by going into a detail of all the occurrences of this sort which might be adduced, but would just mention two striking cases which had come under his own immediate knowledge, selected from his father's case-book; one was that of a lady, who, in the sixth month of her pregnancy, while walking on the South Circular-road, was accosted by a strong sturdy beggerman who solicited alms; she walked on, taking no notice of him, but he, finding his persecution unavailing, drew aside his coat and presented the short stump of an arm that had been removed half way between the shoulder and elbow. The lady, greatly shocked at the sight, got home as fast as she could, but went on well up to her full period; immediately on the birth of the child, however, she asked, with great anxiety, whether there was anything the matter with it. Dr. Beatty's father had heard nothing of the foregoing occurrence, and was of course surprised at the lady's asking the question, but on looking at the child there was found only one arm complete, the other being only a stump, as if after amputation. Such was the story, upon which it was for the society to set what value they pleased. The next case was one recorded several years after, in which the child was born with six fingers on each hand. The lady stated that when about five months pregnant, a person sitting in the same room with her was suddenly seized with epilepsy, and the patient's hands moved so rapidly that the fingers appeared to the lady to be multiplied to an immense number. She could never after banish the vision from her mind, and her child was born with these supernumerary fingers. Here, then, was another remarkable instance to be added to the general mass of facts in relation to this subject. In the case to which he more immediately drew the attention of the society this evening, there existed, as he had before observed, a plurality of children. It was very rarely indeed, he said, that four children are produced at a single birth, so that the present case furnished an additional evidence of the prolific character of the females of this country, although it was stated by Devereux that in America the number of twin births far exceeds those of any other country in the world; while it is maintained by Mr. Collins of this city, that the occurrence of twin births is much more common than in any other country. A note in Ramsbotham's work, however, he thought offered rather a satisfactory explanation of these two conflicting opinions. It is perfectly well known, he says, that America is to a great extent peopled by the Irish, there being of course a mixture from other nations, but the tendency was clearly to a majority of the population being constituted by the Irish. Dr. Beatty had brought with him, he said, drawings of two celebrated monsters formerly exhibited to the society; his object in presenting them now was not, he said, with a view of making much use of them on this occasion, but merely for the purpose of comparison. One of these cases had given rise to the late Dr. Houston's celebrated paper on the means by which the circulation is carried on in monsters generally, and which had been the occasion of a very spirited controversy between him and a gentleman in England. In that case there was no heart, thorax, head, or upper extremities. The other drawing represented a very remarkable specimen of monstrosity by fusion, which had been exhibited to the society by Dr. Speedy some time since, and was, Dr. Beatty observed, as curious an example of this class of monstrosities as ever he had seen, the only trace of anything like limbs being two fin-like processes, but there was in no other way the slightest resemblance to a human being.

Dr. Darby said it had not been his intention to take any part in the discussion, but the occurrence of an instance bearing upon the subject under consideration was just before his mind. About a fortnight since he had been called up about four o'clock in the morning to a twin-case, the second child being a monster, on seeing which the midwife was so frightened that she sent immediately for him. On arriving, he found nothing remarkable in the state of the woman, and on examining the monster, it presented no signs of life; it appeared at first view to have but one head, but on closer inspection two distinct brains were apparent at the back of the head; in the front there was a single forehead, but there was apparently two faces lying together, and united at the chin; the upper lip was perfect; the under one was divided. There was four eyes, and at the upper part of the back there was spina bifida. The woman had previously given birth to several children, and on one occasion to twins; some of the children had evidently a scrofulous tendency, the woman herself being also of an apparently scrofulous diathesis. He was not aware that there had been any mental impression received in this case. The question of mental impressions was undoubtedly a most important one. Mothers were every day to be met with who could hardly be persuaded that their children would not be born with some imperfection from their having themselves received some fright; hence it would be a great matter, he thought, if men in practice would try to do away with these notions; his own impression was, that they were mere superstitions. While assistant to Dr. Macartney formerly, he had often dissected subjects in whom a hand or foot had apparently been lost, but on examination the fœtal rudiments of these limbs, were to be found enveloped in integument at the end of the extremity.

Dr. Power observed, that he thought a great means of settling the question of mental impressions would be the ascertaining at what particular stage and development of the fœtus the impression was received; if, for example, in hare-lip, such impressions were made before the time at which union takes place between the two halves of the upper maxilla, or in the case of the arm, whether the impression had been made previous to the lying down of its rudiments, the influence of these impressions might then be said to be so and so. With respect to the case of the lady six months pregnant, which had been alluded to by his friend Dr. Beatty, it necessarily became a question whether the mental impression produced absorption of the hand and of a great part of the forearm, or whether a long time before, the accident at a very early period, induced an arrest of development, had not arisen from some cause. These, he thought, were two questions worthy of consideration. For himself, he was inclined to the opinion that these cases were rather to be looked on in the light of coincidences than as consequences of such accidents. It would be rather difficult, he thought, to conceive the possibility of absorption of the forearm in Dr. Beatty's case; therefore, the other explanation might be the more feasible one.

Dr. Beatty hoped the society were not of opinion that he was an advocate for the accuracy of the popular belief respecting mental impressions; he merely mentioned these two cases as historical facts, without offering any opinion upon them. It was for the society to give them whatever weight they seemed entitled to. A satisfactory means of accounting for the case of the lady might, he thought, be derived from the opinions of Dr. Montgomery, who, in some very elaborate papers on this subject, attributes the occurrence of amputation in cases of this kind to the formation of certain bands of organized lymph, between which the limb becomes entangled, and absorption effected by the gradual tightening of them. The limb being frequently found in this detached manner at the birth of the child demonstrates the truth of Dr. Montgomery's statements.

Mr. M'Coy took occasion to remark that in the question under consideration, he believed a man might almost throw himself into either scale without losing weight. In perusing the mass of French details of the effects of mental impressions during pregnancy, it would not be easy to predicate whether even a strong mind would incline to an admission of a possible case being made out of cause and effect, or plead mere coincidence for its infidelity. One thing, however, is certain, that women themselves are firm believers, that marks and monstrosities are generally, if not always, owing to physical or mental causes acting from without during utero-gestation; and whether the question is to be solved through physiology or metaphysics—by the transcendentalist or pathologist, it is unquestionably one of much interest, even from the doubts it has caused.

Dr. Mitchell would be very far from denying altogether that mental impressions were incapable of producing any effect, for there were certain circumstances connected with them which it was impossible entirely to overlook. He might remind the society of an acephalous fœtus which he had exhibited to them the winter before last; it was a six months fœtus, the mother while at her washing-tub had a cat thrown on her back by her brother, and the society was acquainted with the remarkable facts that attended the birth of the monster, which lived and breathed for forty minutes. Amongst the very curious points connected with that case, there was an extraordinary development of the platysma. Viewing that one case alone, he contended that some weight should be given to the doctrine.—***Dublin Medical Press.***

